# Towards Biomanufacturing of Cell-Derived Matrices

**DOI:** 10.3390/ijms222111929

**Published:** 2021-11-03

**Authors:** Weng Wan Chan, Fang Yu, Quang Bach Le, Sixun Chen, Marcus Yee, Deepak Choudhury

**Affiliations:** 1 Biomanufacturing Technology, Bioprocessing Technology Institute (BTI), Agency for Science, Technology and Research (A*STAR), 20 Biopolis Way, Singapore 138668, Singapore; chan_weng_wan@bti.a-star.edu.sg (W.W.C.); quang_bach_le@bti.a-star.edu.sg (Q.B.L.); chen_sixun@bti.a-star.edu.sg (S.C.); marcus.a.y.x@gmail.com (M.Y.); 2 Smart MicroFluidics, Singapore Institute of Manufacturing Technology (SIMTech), Agency for Science, Technology and Research (A*STAR), Fusionopolis Way, Singapore 138634, Singapore; fang_yu@simtech.a-star.edu.sg

**Keywords:** cell-derived matrices, extracellular matrix, decellularised extracellular matrix, biomaterials, hydrogels, biomanufacturing, bioink, bioprinting, CDM, dECM

## Abstract

Cell-derived matrices (CDM) are the decellularised extracellular matrices (ECM) of tissues obtained by the laboratory culture process. CDM is developed to mimic, to a certain extent, the properties of the needed natural tissue and thus to obviate the use of animals. The composition of CDM can be tailored for intended applications by carefully optimising the cell sources, culturing conditions and decellularising methods. This unique advantage has inspired the increasing use of CDM for biomedical research, ranging from stem cell niches to disease modelling and regenerative medicine. However, while much effort is spent on extracting different types of CDM and exploring their utilisation, little is spent on the scale-up aspect of CDM production. The ability to scale up CDM production is essential, as the materials are due for clinical trials and regulatory approval, and in fact, this ability to scale up should be an important factor from the early stages. In this review, we first introduce the current CDM production and characterisation methods. We then describe the existing scale-up technologies for cell culture and highlight the key considerations in scaling-up CDM manufacturing. Finally, we discuss the considerations and challenges faced while converting a laboratory protocol into a full industrial process. Scaling-up CDM manufacturing is a challenging task since it may be hindered by technologies that are not yet available. The early identification of these gaps will not only quicken CDM based product development but also help drive the advancement in scale-up cell culture and ECM extraction.

## 1. Introduction—Cell-Derived Matrices (CDM) and Their Applications

### 1.1. What Is CDM?

Animal cells are strictly dependent on their surrounding ECM for structural and biological supports from cellular adhesion to growth and differentiation. The ECM consists of two main classes of macromolecules: fibrillar proteins (such as collagens, laminins, and fibronectins) and proteoglycans (such as aggrecan, perlecan, and versican) [1]. These molecules provide most of the needed signals for cellular homeostasis and development [2], and thus the cell-free ECM, dubbed decellularised ECM (dECM), is a highly bioactive material [3,4]. The removal of cells is necessary to reduce the immunogenicity of the allo- and xeno-grafts. Traditionally, dECM is obtained from cadavers where the tissue or organ of interest (such as skin, cartilage, or bone) is harvested and decellularised [4,5,6]. More recently, the advent of in vitro cell culture has led to the process of growing animal tissue in the lab using only stem cells originating from animals. The cultured tissue can then be used either with living cells (e.g., cultured skin autograft) or without the cells, i.e., cell-derived matrices (CDM) [7].

### 1.2. CDM vs. dECM

CDM and dECM are both animal cell ECM, with the former derived from lab-grown cells/tissues and the latter from actual animals. Both are processed with similar decellularisation methods using various types of detergents and reagents [4,6,8]. The key differences are the obtained quantity and characteristics of the final ECM (Table 1). CDM can have a very similar chemical composition to dECM of the same tissue origin, but it is difficult to make CDM with the same physical characteristics of the native tissue, such as structural organisation. For instance, a full organ’s dECM scaffold can be simply procured through careful and thorough perfusion decellularisation [6,9,10]. CDM often does not possess any structural or mechanical trait apart from its chemical composition. However, it can be argued that CDM need not possess any of the physical property, especially when it is intended for application where ECM macrostructure is unnecessary.

The major benefit of CDM over dECM is tunability. In the CDM manufacturing process, the lab-grown tissue is at the centre of the operation. Cell sources and culture methods can be engineered to tailor the secreted ECM for specific actions. For example, genetically engineered cells can be utilised to produce specific engineered matrix proteins [1]. CDM projects involve optimising multiple factors, rapidly producing ECM prototypes and testing them for the intended application. The obtained results will be feedbacked to the optimisation process. This allows CDM to be tuned extensively for its intended purpose. For dECM, the only applicable change is the decellularisation methods, and, in fact, batch-to-batch variations due to different animal sources can mean limited control over the eventual biomaterial.

Finally, while dECM often faces sustainability (not enough animal source) and consistency (animal-to-animal variability) issues [11], a fully optimised CDM manufacturing process can theoretically produce an indefinite quantity of ECM at very high consistency.

### 1.3. Applications of CDM

Like dECM, CDM has broad application in biomedical research, tissue engineering and regenerative medicine. Such applications in research include mechanistic studies of cell–ECM interaction and the effects on physiological or pathophysiological development of cells/tissues [7,12,13]. In medicine, dECM and CDM have been extensively used as biomaterials for constructing grafts (such as bone graft and skin graft) and medical devices (as a base component in bioinks for 3D bioprinting) [14,15,16,17]. Most recently, the nascent field of cultured-meat manufacturing is actively seeking suitable bioactive scaffolds [18,19,20,21]. CDM could be just the needed scaffold for the industrial cultivation of animal tissue. Moreover, the animal-free origin of CDM fits perfectly with the sustainability theme of the cultured meat industry [22].

With increasing research outputs, CDM has the potential to become incredibly important for the biomanufacturing industry. Despite this, little work has been done to prepare for the next wave of the CDM production frenzy. Scaling up and industrial production has been grossly overlooked in the early stages of CDM research projects, which will certainly impede product development in the long term. This review aims to introduce the current methods for CDM production/characterisation and the existing technologies for scale-up production. We will also highlight the considerations and challenges faced in adopting these scale-up technologies.

## 2. Existing Methods of CDM Production/Characterisation

### 2.1. A Basic Protocol for CDM Production

Depending on the application, CDM can be produced on 2D or 3D cell culture conditions. There are four main steps in the CDM production process: cell expansion, seeding, matrix generation/deposition, and decellularisation/harvest.

Cell expansion: The cells for CDM production that originated from animal tissues must undergo isolation, characterisation, initial expansion, and cryopreservation (cell banking) for future use [23]. Each batch of CDM production starts with frozen vials of cells, which are extensively expanded to obtain a sufficient cell number for manufacturing. During expansion, it is important that the cells maintain their stemness and minimise senescence, both of which may affect subsequent ECM production [24].

**Figure 1:**
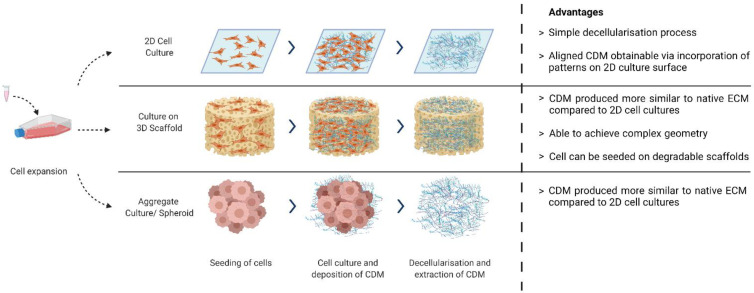
Different methods of CDM production. Created with BioRender.com on 1 October 2021.

Cell Seeding: Expanded cells can be seeded on either 2D surfaces [3], 3D scaffolds [15,25], or as cell aggregates [26,27] (Figure 1). The choice depends on the desired application: whether the CDM will be used as tissue-engineered constructs or as bulk materials for further processing (such as a bioink component). Examples of cells used are fibroblast [3,7], chondroblast [27], osteoblast [28], and stem cells [13,24].

CDM matrix generation/deposition: The most important step in CDM production, where the most amount of effort is spent, is in creating the ideal culture condition that stimulates the desired ECM production. This deposited ECM is composed of a complex and highly organised macromolecular network of biomolecules and cell signalling factors. By systematically optimising the intrinsic (e.g., by genetically modifying cells) and extrinsic factors (e.g., scaffolds, media, growth factors, and physical stimulants), it is possible to achieve the desired physical and biochemical properties for the final ECM product.

Decellularisation/CDM harvest: During decellularisation, the cellular component of the cultured tissue is removed with the use of physical, chemical, or enzymatic means. This is critical not only to reduce the immunogenicity of the material for implantation in the body [29], but also to preserve its physical structure and biochemical component [6].

### 2.2. Characterisation of CDM

The characterisation is done to verify the presence of the desired features and composition of the produced CDM. Stringent characterisation serves as feedback in the optimisation of the manufacturing process, as well as quality control of the CDM product. The broad categories of biomaterial characterisation can also be applied to CDM, including physical, chemical, mechanical, surface, in vitro, and in vivo characterisation [30]. However, choosing the right characterisation tools depends again on the final product application, and careful selection will save both time and resources. Below are some of the useful characterisation techniques that have been used for CDM.

Biochemical analyses target the biochemical composition within the CDM. For instance, total collagen content can be estimated by hydroxyproline assay; total glycosaminoglycans content by GAG assay; and residue genetic material by DNA quantification kits. More specific molecules such as specific collagen types, fibronectin, and certain growth factors can be identified or quantified by techniques such as Western blot and enzyme-linked immunosorbent assay [7,28,31,32]. Certain parameters are important for CDM, such as maintaining a DNA content below 50 ng/mg to reduce the risk of immunogenic response [5].

Immunostaining, in combination with histology and imaging techniques, allows for identification and structural visualisation of the molecules of interest. This is useful to verify the position of target molecules (such as fibronectin and collagen) in the CDM where the directional organisation is desired [33].

The proteomic analysis provides highly accurate and detailed information on the composition of CDM proteins. For instance, through mass spectroscopy-based techniques, the composition of CDM produced from different stem-cell types can be distinguished [34]. Additionally, mass spectrometry can be used as a quality control assay to identify residual detergent fragments in the decellularised tissue [35].

The CDM ultrastructure can be viewed through methods such as atomic force microscopy (AFM), scanning electron microscopy (SEM), and transmission electron microscopy (TEM). AFM is used to analyse the surface roughness of the CDM, while SEM is used to observe the sectional and surface structure of the CDM. Additionally, TEM is used to observe the assembly of collagen fibre arrangement [36].

Mechanical analysis of CDM can be done using a mechanical tester or AFM. Strips of CDM can be attached to a mechanical tester where tensile strength is measured [37]. Alternatively, by utilising an AFM in contact mode to obtain force–distance curves, the elastic modulus of the CDM can also be obtained [38].

### 2.3. Tunable Properties of CDM

Unlike animal-source-derived dECM, CDM is manufactured entirely in the lab from animal-origin stem cells. The properties and composition of CDM can be changed extensively by altering the intrinsic (choice of cells and genetic engineering) and extrinsic factors (use of additives and culture conditions).

The cell source will singly determine its CDM composition. For example, the proteomic characterisation of CDM extracted from bone-marrow-derived mesenchymal stem cells (MSC), adipose-derived MSC, and neonatal dermal fibroblast showed a distinct signature for each of them [34].

A common additive used to increase the collagen content in CDM is ascorbic acid (vitamin C). It is an essential cofactor in collagen synthesis and doubles as an antioxidant that scavenges free radicals in the culture [39]. Additionally, growth factors and matrix proteinase inhibitors have been shown to increase collagen content [31]. The use of synthetic macromolecular globules (such as Ficoll and polyethene glycol) for macromolecular crowding can enhance matrix deposition as well as induce alignment [40].

Cell culture conditions such as hypoxia, substrate stiffness, and topography will also have an influence on CDM composition. Hypoxia was shown to improve the deposition of fibrillar collagen by fibroblasts [41]. The stiffness of the substrate on which cells were cultured influences their phenotype, which in turn impacts the composition and properties of the ECM [42]. Similarly, the substrate topography will also determine the structural organisation of the ECM. The use of micro-moulds and grooves has been shown to result in isotropic fibre structures and architecture in the deposited ECM [33,43].

## 3. Scale-Up Technologies for CDM Production

To date, most CDM productions are on a laboratory scale, yielding micrograms to grams of materials from individual cultures [1,33]. Scaling up CDM production can be tackled by increasing the yield and reducing the cost of production. Several scale-up strategies will be discussed below.

### 3.1. Scaling up Cell Culture Platforms

**Figure 2:**
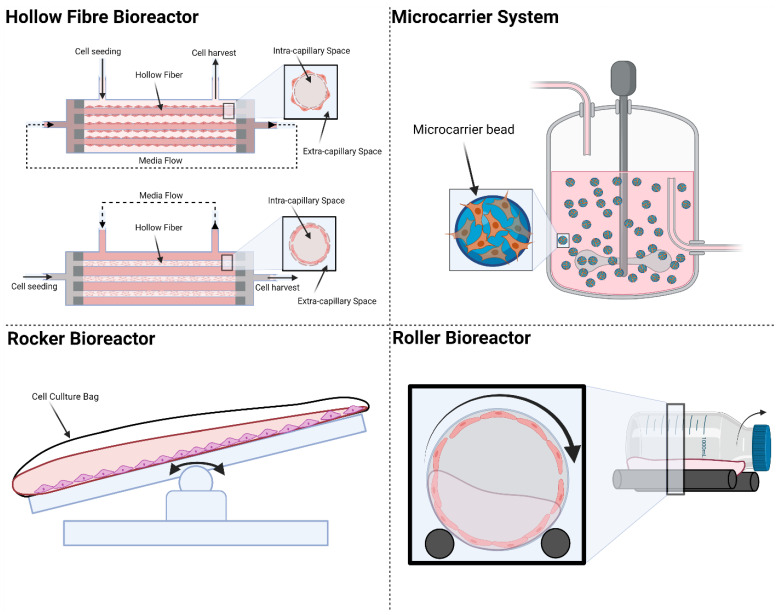
Existing cell culture platforms: Hollow Fibre bioreactors, microcarriers, rocker bioreactors, and roller bioreactors. Created with BioRender.com on 1 October 2021.

One of the costliest steps in CDM production is cell expansion, which is traditionally done in cell culture flasks and plates for adherent cells. Cell expansion on flat-plate culture platforms is laborious and costly. One single batch of industrial CDM manufacturing can easily consume billions of cells, making cell expansion a financially critical step [44]. Industrial cell culture platforms need to provide the high throughput requirement while maintaining the consistency of the culture condition. Existing cell culture platforms that can be adopted to scale up CDM production are shown in Figure 2.

#### 3.1.1. Hollow Fibre Bioreactors

Hollow fibre (HF) bioreactors consist of cylindrical compartments packed with porous hollow fibres. The culture medium is connected to the HF reactor to flow through the fibre or the whole compartment or both according to application. Cells can be seeded and cultured inside or outside the surface of the fibres. The large surface area of the HF allows high cell densities on the order of 10 million cells per mL to be achieved [45,46,47]. The pore size of the semi-permeable fibres can be customised to determine if cells can pass through or be restrained by the membrane. If the cells are seeded inside the hollow fibres, the medium will be perfused outside the fibres. This flow configuration is called intra-capillary seeding; in this case, the medium is perfused outside of the fibres. HF bioreactors can provide a large increase in surface-to-volume ratio for cell culture compared with static cultures but has shortcomings in monitoring the cells with imaging methods. The design of the HF mimics blood vessels in facilitating nutrient supply and waste removal. Gas exchange is achieved by simple diffusion across the intra-capillary and extra-capillary spaces. Some of the commercial sources for HF bioreactors include FibreCell [48], NxStage [49], and Quantum^®^ Cell Expansion System [50].

#### 3.1.2. Microcarriers

Microcarrier is another commonly used platform to scale up adherent cell production in the industry. The microcarriers are usually 100–300 μm in diameter and can provide a much larger surface area for cell growth as compared with 2D culture platforms. The microcarriers can be surface-modified and functionalised with different coating materials depending on the cultured cell types for optimal cell attachment. There are two categories of microcarriers: solid spherical microcarriers and porous microcarriers. For solid microcarriers, cells are only attached to the outer surface of the microcarriers. Porous microcarriers can be fabricated with cellulose or gelatine to form the hollow structure, which allows cells to migrate and attach inside the microcarriers. Solid microcarriers are commonly made of dextran [51] and polystyrene [52]. They have been used for scaling up culture and expansion of mesenchymal stem cells, induced pluripotent stem cells, and embryonic stem cells [53,54]. They are also extensively used together with stirred tank bioreactors. The microcarriers are suspended in culture medium and moved around by the stirring motion inside the bioreactor. Commercially available porous carriers include CultiSpher™ [55] and Cytopore™ [56]. These systems claim to improve cell density and protect the cells from shear stress when cultured inside stirred tank bioreactors.

For microcarrier-based cell culture platforms, harvesting cells and the secreted CDM can be a challenge since the enzymes used for cell detachment can adversely affect the quality of the CDM. Another challenge is the hydrodynamic profile of the bioreactor: when not regulated properly, the microcarriers may collide with each other resulting in damage to the cells.

#### 3.1.3. Rocker Bioreactor

In a rocker bioreactor, the culture medium is set in gentle motion by the periodic movement of the rocking platform. This motion allows for good mass exchange between cells and culture medium. The rocker bioreactor can be easily scaled up and automated; this has been demonstrated for large scale cell culture with 500 L of volume [57]. Rocker-based bioreactor platforms have been commercialized and some examples are BioWave^®^ [58], Wave Bioreactor™ [59], Biostat^®^ CultiBag RM [60], Tsunami^®^ Bioreactor [61], CELL-tainer^®^ Bioreactor [62], and Undertow Bioreactor (WUB) [63]. Like the stirred tank bioreactors, rocker-based platforms can be combined with microcarriers to further increase the surface to volume ratio for cell culture.

#### 3.1.4. Roller Bioreactor

Roller bioreactors are also called rotating wall vessel bioreactors. The roller bioreactor system usually consists of cylindrical bottles placed on roller racks inside the incubators. These bottles are rotated in cylindrical motion. During the culture, the cells are either submerged in the culture medium or exposed to gas inside the roller bottle [64]. The roller bioreactor configuration is best suited for adherent cells and can also be combined with microcarriers. Large number of bottles can be used to scale up the production. However, the size of each bottle is usually limited, which can complicate the operating process [65].

### 3.2. Increasing CDM Yield through Small-Molecule Intervention or Genetic Engineering

#### 3.2.1. Small-Molecule Intervention

One way to increase CDM yield is to induce higher ECM secretion by using pharmaceutically active small molecules. Pharmaceutical research has elucidated various regulatory pathways for ECM deposition from physiological and pathophysiological processes [66]. For instance, studies of ECM-remodelling in diseases have revealed that an increase in metalloproteinase expression induces collagen loss and ECM breakdown [67,68] and the activation of the TGFβ pathway is a well-known cause for excessive ECM production in fibrosis [69]. Another example is the increased ECM production in cancer cells as the disease progresses; the epithelial-to-mesenchymal transition [70,71] and the acquisition of chemoresistance [14,72]. By high-throughput screening, novel small molecules are increasingly discovered that are capable of inducing or inhibiting these pathways.

The small molecule approach has proven a useful tool for tissue engineering due to its cost-effectiveness, stability, time of action, and reversible effects. In choosing small molecules to scale up CDM production, the main ECM component of interest must be identified, followed by searching for available small molecules capable of increasing its expression through online databases such as the National Institute of Health (NIH)’s Library of Integrated Network-Based Cellular Signatures (LINCS) Programs [73] and Drugbank [74]. It is crucial to test the actual CDM yield vs. cytotoxicity effect of the compounds to establish suitable working concentrations. Furthermore, quality control of the final CDM product must report the presence of any trace amount of the small molecules that might affect its downstream application.

There are two main drawbacks of using the small-molecule approach. First, unwanted changes may occur in the CDM composition due to changes in cell state upon treatment or off-target effects of the molecules. This can be prevented by careful selection of the drug compounds and their working concentration based on the full characterisation of the resulting CDM. Second, at times there is simply no small-molecule available that can perturb the targeted ECM component of interest. In this case, genetic engineering may be the next consideration.

#### 3.2.2. Genetic Intervention

As CDM is the product of in vitro cell culture, the fundamental scale-up capacity depends on the possession of stable cell lines capable of secreting good quantity and quality of ECM. Developing a suitable cell line is a necessary task since quantity and consistency are crucial for an industrial manufacturing pipeline. Existing strategies include genetic perturbations such as stable overexpression of ECM proteins, deletion/silencing of matrix metalloproteinases, and activation of targeted pathways.

Lentiviral transduction is a common method used to generate stable cell lines through the integration of vectors coding for mRNA or shRNA that induce overexpression or silencing of the targeted proteins. Recent gene-editing techniques, such as clustered regularly interspaced short palindromic repeats (CRISPR), are very efficient at specific deletion/addition of targeted DNA in the genome, resulting in permanent silencing/overexpression of the targeted proteins [75].

Compared to small-molecule intervention, genetic engineering to induce specific proteins is a more straightforward scale-up approach. However, genetic engineering might not be easily performed on certain cell types, especially primary cells [76]; moreover, direct overexpression of large ECM components such as collagen and fibronectin would not be efficient. Manufacturers would have to consider the cell type, desired CDM composition, targeted functionality, and cost of perturbation to make a sound decision on which methods best suits their needs.

Table 2 summarises the various approaches for CDM manufacturing.

## 4. Discussion

### 4.1. Considerations When Scaling up CDM Manufacturing

**Figure 3:**
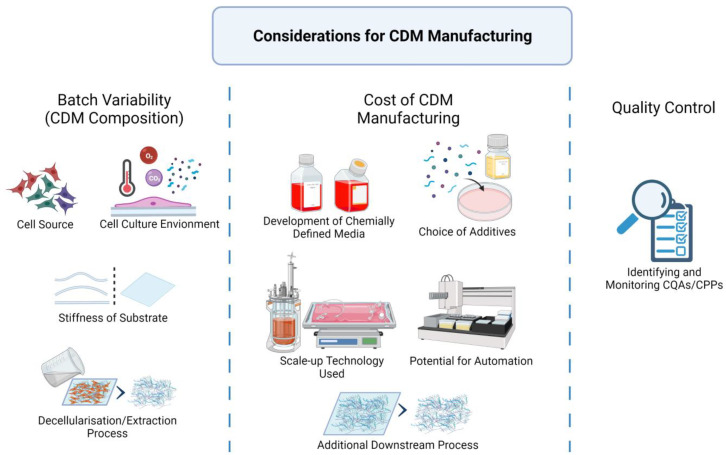
Considerations when scaling up CDM manufacturing. Created with BioRender.com on 1 October 2021.

The scaling of CDM production from laboratory to manufacturing has multiple considerations, as seen in Figure 3: batch variability, manufacturing cost, and quality control.

Batch variability refers to the consistency of the CDM composition. Any change to the intrinsic and extrinsic factors during manufacturing will result in the deviation of the CDM product. The first consideration in scaling up production is to fix and secure all the various factors: cell sources, medium components, bioreactors, chemicals, and processing methods.

Cell sources such as primary cells and cell lines play a role in the batch variability of CDM. Primary cells are variable by nature; thus, appropriate pre-characterisation and cell-banking strategies must be implemented to maintain the required standard. Without genetic modification, only stem cells derived from primary sources have the capability to undergo sufficient cell division for CDM manufacturing at scale. For example, embryonic stem cells are often considered immortal in culture [77], mesenchymal stem cells can have between 13 and 25 population doublings [78], and epidermal stem cells can have up to 115 population doublings [79] before undergoing senescence. For industrial manufacturing, maintaining a stem-cell source is a tremendous task, considering the logistics of obtaining donor cells, isolation, ‘stemness’ maintenance, banking and quality assurance.

Cell line, or genetically engineered cells, is a much better option for industrial CDM production due to their capability of being maintained for a very long period. However, cell lines may still lose their special characteristics and need regular quality control and characterisation [1]. Numerous mammalian cell lines have been developed for the industrial production of biotherapeutic proteins, notably the Chinese hamster ovary (CHO) expression systems [44]. However, more development is needed to express larger ECM proteins using these systems.

Cell culture conditions (such as temperature, oxygen, additives, and substrate scaffolds) directly affect cell growth and ECM deposition and should be kept under strict control [39,40,41,42]. Finally, the decellularisation/extraction process should also be standardised or automated, as any alteration in methods, chemicals, or perfusion rate will result in changes to the CDM product [29].

Manufacturing cost includes the costs of research/development and of production. The cost of developing chemically defined media needs to be considered. Optimising culture media is a costly and time-consuming endeavour due to the complexity of potential formulations. However, a well-defined medium may be invaluable when it comes to reducing the usage of expensive growth factors and serum [80,81,82]. Another factor is the choice of scale-up cell culture platforms, the costs of which depend on the medium consumption over CDM yield and whether the technology requires extra downstream processing steps (such as the removal of microcarriers). Finally, the potential for automation should also be considered. Automation will reduce manual handling and hence labour cost. Plus, automation will mitigate human errors, resulting in more consistent quality and improved manufacturing performance [83].

Quality control ensures the final product consistently meets the designed specifications, which is the hallmark of any industrial process. This can be achieved by identifying and monitoring critical quality attributes (CQAs) and critical process parameters (CPPs). CQAs is defined as “a physical, chemical, biological characteristic that should be within an appropriate limit, range, or distribution to ensure the desired product quality”. CPPs refer to the parameters in the production process that impact the CQAs, which in turn affect the quality of the final product. Hence, by establishing the CQAs and CPPs, manufacturers would ensure consistency in the final products [84].

### 4.2. Challenges of Existing Scale-up Technologies for CDM Production

Existing scale-up technologies address the issue of nutrition transfer with a high surface area-to-volume ratio. However, they do not address other factors, such as the effect of dynamic culture and additional steps for downstream processing.

The dynamic nature of the platforms (e.g., perfusion rate for the hollow fibre bioreactors, sparging rate for the microcarrier bioreactor vessels, revolutions per minute for the roller bottles, rocking cycles per minute for the rocker bioreactor) will influence the composition of the final CDM products. Since the effect of dynamic culture on CDM production is not well-established, it is important to carefully characterise the obtained CDM with each new scale-up technology prior to its adoption for large-scale production.

Originally, most scale-up platforms were developed for the extraction of cells or biologics (such as soluble proteins), not the whole ECM with abundant insoluble macromolecules. As such, CDM manufacturers would need to develop extra downstream processes in order to harvest CDM from the existing systems (e.g., removal of CDM from the hollow fibre channels, microcarriers, or vessel surface). This extra processing must be carefully designed to avoid altering the desired CDM composition or contaminating the products with unwanted residual chemicals.

## 5. Conclusions

The biomimicking nature of CDM makes it a valuable biomaterial for a wide range of applications, from fundamental research to clinical medical devices, cell therapy, and even potential tissue-engineering in the cultured meat industry. Most CDM projects are often in R&D at a laboratory scale; the idea to scale up CDM production only comes at the later stages of product development. This paradigm should be rethought, as we have highlighted here. While certain scale-up technologies exist, there is no guarantee that they can achieve high throughput for CDM production. Every parameter (cell lines, medium components, and scaffolds, to name a few) is better solved as early as possible in the product development process. Tremendous effort is needed to scale up CDM production, considering yield, quality, and economic factors. The development of automation technology, including inline quality control and monitoring, will be much needed for future CDM manufacturing.

## Figures and Tables

**Table 1:** Decellularised extracellular matrices (dECM) vs Cell-derived Matrices (CDM).

	dECM	CDM
Advantage	a. Possess chemical/physical composition of the native tissue b. Proven technology	a. Tunable composition for intended applications b. Consistent quality c. Lower immunogenicity d. Sustainable, reduce animal usage
Disadvantage	a. Shortage of animal/donor tissue b. Batch to batch variability c. Higher immunogenicity d. Depend heavily on animal husbandry/human donor	a. Difficult to achieve native tissue structure b. Less characterised for various tissue types c. Challenging to scale up

**Table 2:** Summary of approaches for CDM manufacturing.

Approach	Description	References
Lab-Scale		
2D Cell culture	Cells are cultured on 2D surface, and the deposited CDM are decellularised and used.	[3,7,13,33,34,36,38,43]
Culture on 3D scaffolds	Cells are cultured on 3D scaffolds (natural/synthetic); the resulting tissue can be used as tissue-engineered constructs (live/decellularised), or CDM can be extracted (decellularised).	[11,15,28,37] [12,31]
Scaffold-free culture	Cells are cultured as 3D aggregates/spheroids in a scaffold-free environment. The aggregates are then decellularised to obtain CDM.	[26,27]
Scale-Up		
Hollow Fibre Bioreactors Microcarriers Rocker bioreactor Roller bioreactor	Scale-up cell culture platforms that significantly increase adhesion cell density by optimising cell attachment surface area, medium, and gas flow; suitable for improving CDM yield.	[45,46,47,48,49,50] [53,54,55,56] [57,58,59,60,61,62,63] [64]
Small-molecule intervention	Utilising small molecules to induce higher ECM deposition resulting in higher CDM yield.	[66]
Genetic intervention	Genetically modifying cells to stably overexpress desired ECM components and increase CDM yield.	[75]

## Data Availability

Not applicable.
